# Differential Recruitment of Parietal Cortex during Spatial and Non-spatial Reach Planning

**DOI:** 10.3389/fnhum.2017.00249

**Published:** 2017-05-09

**Authors:** Pierre-Michel Bernier, Kevin Whittingstall, Scott T. Grafton

**Affiliations:** ^1^Département de Kinanthropologie, Université de Sherbrooke, SherbrookeQC, Canada; ^2^Département de Radiologie Diagnostique, Université de Sherbrooke, SherbrookeQC, Canada; ^3^Brain Imaging Center, Department of Psychological and Brain Sciences, University of California, Santa Barbara, Santa BarbaraCA, USA

**Keywords:** arm reaching movements, parieto-frontal network, movement planning, EEG, fMRI

## Abstract

The planning of goal-directed arm reaching movements is associated with activity in the dorsal parieto-frontal cortex, within which multiple regions subserve the integration of arm- and target-related sensory signals to encode a motor goal. Surprisingly, many of these regions show sustained activity during reach preparation even when target location is not specified, i.e., when a motor goal cannot be unambiguously formed. The functional role of these non-spatial preparatory signals remains unresolved. Here this process was investigated in humans by comparing reach preparatory activity in the presence or absence of information regarding upcoming target location. In order to isolate the processes specific to reaching and to control for visuospatial attentional factors, the reaching task was contrasted to a finger movement task. Functional MRI and electroencephalography (EEG) were used to characterize the spatio-temporal pattern of reach-related activity in the parieto-frontal cortex. Reach planning with advance knowledge of target location induced robust blood oxygenated level dependent and EEG responses across parietal and premotor regions contralateral to the reaching arm. In contrast, reach preparation without knowledge of target location was associated with a significant BOLD response bilaterally in the parietal cortex. Furthermore, EEG alpha- and beta-band activity was restricted to parietal scalp sites, the magnitude of the latter being correlated with reach reaction times. These results suggest an intermediate stage of sensorimotor transformations in bilateral parietal cortex when target location is not specified.

## Introduction

To plan goal-directed arm reaching movements, the brain must transform goal-related sensory information into an appropriate pattern of motor commands. This is achieved by integrating the sensory signals that define the position of the goal and those that define the position of the arm. Studies in non-human primates have highlighted the important role of specific regions of the dorsal parieto-frontal cortex in this process, namely the parietal reach region (PRR) (Snyder et al., 1997; Galletti et al., 1999; Andersen and Buneo, 2002; Gail and Andersen, 2006), which encompasses the medial intraparietal sulcus (MIP) and area V6A, as well as the dorsal premotor cortex (PMd) (Cisek and Kalaska, 2002, 2005; Hoshi and Tanji, 2006). These regions tend to be more active during the planning of arm reaching movements as compared to the planning of movements of other effectors, such as saccadic eye movements (Snyder et al., 1997; Premereur et al., 2015). They are also thought to be more functionally linked to the contralateral effector. For instance, they show more sustained activity before movements of the contralateral arm as compared to the ipsilateral arm (Snyder et al., 1997; Hoshi and Tanji, 2006; Chang et al., 2008). In addition, PRR firing correlates better with reach RTs of the contralateral arm (Chang et al., 2008), and inactivation of this region mainly affects reaching movements of the contralateral arm (Yttri et al., 2014). Finally, planning-related activity of most cells in these regions is tuned to the direction of an impending reaching movement during planning (Buneo et al., 2002; Cisek and Kalaska, 2002, 2005; Scherberger et al., 2005; Pesaran et al., 2006). Together, these data suggest that these regions encode not only the visuospatial location of a target, but integrate it with arm-related signals to form a motor goal (i.e., reach direction between the arm and the target).

These observations have been extended to human reach regions based primarily on neuroimaging. Indeed, the human dorsomedial PPC and PMd specifically take part in arm-target integration for the planning of reaching movements (Connolly et al., 2003; Beurze et al., 2007, 2009; Fernandez-Ruiz et al., 2007; Van Der Werf et al., 2010; Gallivan et al., 2011; Heed et al., 2011; Vesia and Crawford, 2012; Leone et al., 2014; Cappadocia et al., 2016). These regions are more active when both the arm and the target are known in advance as compared to when only one source of information is provided (Beurze et al., 2007; Heed et al., 2011; Bernier et al., 2012). They also show more robust responses contralateral to both the arm to be used and the hemispace in which a target is presented (Beurze et al., 2007), suggesting the specification of the two sources of information. Finally, using repetition suppression (Bernier and Grafton, 2010; Fabbri et al., 2010) or multivoxel pattern analysis (MVPA) (Gallivan et al., 2011; Barany et al., 2014; Gertz et al., 2017), activity in these regions has been shown to be modulated as a function of the direction of an upcoming reaching movement, demonstrating that they take part in the encoding of a motor goal.

Interestingly, during a preparatory delay period, neuronal activity in non-human primates is also commonly observed in the dorsal parieto-frontal network in the absence of target visuospatial information. For instance, neurons in the monkey PRR and PMd show a tonic increase in spiking activity during a delay period in which the animal is instructed to prepare a reaching movement, but before a target is yet specified (Calton et al., 2002; Hoshi and Tanji, 2006; Snyder et al., 2006). Given that these cells are not modulated to the same extent during saccade preparation (Calton et al., 2002), these results have been interpreted as evidence for a reach-specific recruitment and not merely as reflecting general arousal or vigilance. Interestingly, the heightened baseline activity in PRR during non-spatial reach preparation has been shown to better predict reach RTs than when a motor goal can be specified in advance (Snyder et al., 2006), suggesting that this form of preparation is tightly coupled to the dynamics of reaching. In humans, the PPC and PMd also show sustained activity when preparing to reach even though a target is not specified (Beurze et al., 2007; Gertz and Fiehler, 2015). More recently, Gertz et al. (2017) used MVPA during a delayed pro-/anti-reach task, and demonstrated that when there is uncertainty regarding the upcoming direction of a reach, the PMd (but not the PPC) differentiates between the possible target locations during reach preparation. They interpreted these data as reflecting the active maintenance of the coordinates of the potential targets.

In spite of these insights, the functional contribution of non-spatial reach preparatory activity in the parieto-frontal network remains largely unresolved. One possibility is that it reflects processes that facilitate the upcoming reaching movement, without necessarily encoding a motor goal *per se* (e.g., maintenance of target coordinates; Gertz et al., 2017). Another possibility is that it reflects the simultaneous encoding of multiple motor goals, consistent with recent models suggesting that action selection is achieved by competitive interactions between co-existing motor goals (Gold and Shadlen, 2007; Cisek and Kalaska, 2010; Christopoulos et al., 2015).

In this light, the goal of the present study was to compare neural activity in the parieto-frontal network during reach planning when upcoming target location is either specified in advance or not. The present work sought to expand upon previous studies in two main ways. First, EEG was used in addition to functional magnetic resonance imaging (fMRI) to characterize the temporal dynamics of arm-target integration at the population-level, especially in the alpha- (8–12 Hz) and beta-bands (15–30 Hz), which are thought to mediate action preparation in both humans (Tzagarakis et al., 2010; Van Der Werf et al., 2010; Tan et al., 2013; Turella et al., 2016) and monkeys (Scherberger et al., 2005; Stetson and Andersen, 2014). Second, all analyses assessing the influence of spatial cueing were carried out on neural activations that survived a contrast between a reaching task and a finger movement task. This allowed us to investigate reach-related processes more directly, and to control for non-specific factors that also engage the parieto-frontal network, namely low-level sensory processing, visuospatial attention (Colby and Goldberg, 1999) and signals related to the timing of movement onset (Cisek et al., 2009; Saleh et al., 2010; Kaufman et al., 2016).

## Materials and Methods

### Participants

Sixteen right-handed participants (8 males, age range 19–28) took part in the fMRI experiment, which was collected at UCSB. The fMRI data from one participant were discarded due to technical difficulties. Fourteen right-handed participants (8 males, age range 19–28) took part in the EEG experiment, which was collected at the Université de Sherbrooke. All participants gave informed written consent to the experimental protocol, which was approved by the Human Participants Committee, Office of Research, University of California Santa Barbara, or the ethical committee of the Centre Hospitalier de l’Université de Sherbrooke (CHUS). All had normal vision and no history of neurological disease or psychiatric disorders. They were paid for their participation in the study.

### Apparatus

The same task was carried out in the fMRI and the EEG experiments. Since some details regarding the apparatus differed slightly across experiments to optimize the recording of the two datasets, the apparatus is presented separately for fMRI and EEG. Still, the critical features of the task were equivalent.

### fMRI

Participants were positioned in the scanner with their head and neck padded with foam to prevent motion. They wore a set of headphones for ear protection and noise cancelation. Visual stimuli were presented on a custom-built board made of thin opaque fiberglass that rested on participants’ abdomen, which participants looked at through a set of mirrors (distance of the board with respect to the eyes ∼35 cm). Participants were strapped to the table at the level of the chest to prevent excessive movement. Their reaches were done in total darkness; hence participants could not see their reaching hand at any point.

**FIGURE 1:**
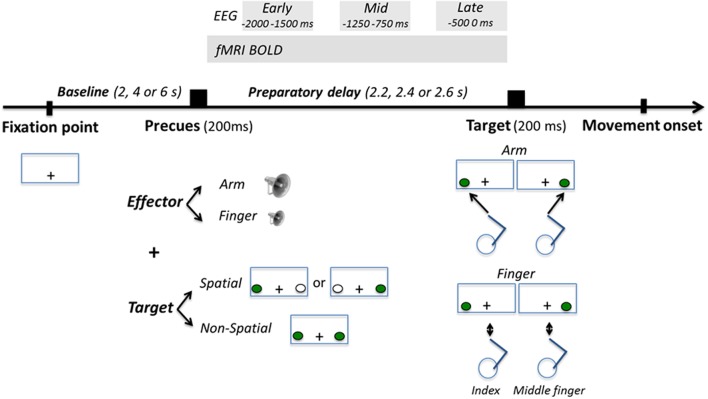
**Task description**. Participants’ task was to respond as fast and accurately as possible to the appearance of a visuospatial target presented left or right of gaze, either by producing a right arm reaching movement toward it, or by producing a finger movement with their right index or middle finger depending on the laterality of the target (left target = index; right target = middle finger). Participants were precued as to the effector to be used with an auditory tone (high or low pitch for arm or finger, respectively) presented 2.2, 2.4, or 2.6 s prior to target onset. At the same time, participants were also precued as to the position of the upcoming target. In the Spatial condition, the left or the right target was presented in green for 200 ms, giving participants advance knowledge as to the position of the upcoming target. In the Non-Spatial condition, both targets were presented in green for 200 ms, giving participants no advance knowledge as to the position of the upcoming target. Neural activity during the preparatory delay was measured with BOLD and electroencephalography (EEG).

The starting point was located perpendicular to the board, 10 cm below the fixation point. It consisted of an MR-compatible button box with two switches that participants pressed with their right index and middle fingers between each movement. The switches required minimal finger force to be depressed. Light emitting diodes (LEDs), which served as a fixation point and targets, were mounted on the stimuli board (**Figure 1**). They were very dim so as not to light up the inside of the bore. The fixation point consisted of a white cross-hair positioned at the center of the board (0° with respect to participants’ body midline). Two targets were used. They were green and could appear at one of two locations, either 5 cm to the left of the fixation point (left target) or 5 cm to its right (right target). Participants were required to gaze at the fixation point throughout the entire experiment; hence reaches were always performed toward targets viewed in the peripheral visual field. The visual angle subtended by the targets from the fixation point was ∼8°. The amplitude of the reaches was short (∼10 cm) so that they could be accomplished mostly through wrist rotation, thereby minimizing motion of the upper arm. Yet participants physically displaced their hand to touch the targets, justifying the use of the term “reaching” instead of “pointing” [i.e., angling the finger in the direction of the target without touching it (Culham et al., 2006)].

### Electroencephalography (EEG)

Participants were seated in front of an experimental setup which consisted of a computer monitor, a semi-reflective mirror, a table and a starting base. The computer monitor (20-inch Dell P1130; resolution: 1024 × 768; refresh rate: 150 Hz) was mounted face down 29 cm above the horizontal semi-reflective mirror, projecting the fixation point and targets onto the mirror. The mirror itself was mounted 29 cm above the table. With this setup the fixation point and the targets appeared to be projected directly onto the surface of the table. The starting base consisted of a button box with two switches that participants pressed with their right index and middle fingers between each movement. The switches required minimal finger force to be depressed. The starting base was located 15 cm in front of participant’s chest directly along their body midline. The fixation point consisted of a white cross-hair also positioned along the midline 25 cm in front of the starting base. The targets were green and could appear at two locations, either 7.25 cm to the left (left target) of the fixation point or 7.25 cm to its right (right target). The visual angle subtended by the targets from the fixation point was ∼7°, so as to approximate that in the fMRI experiment. Participants were required to gaze at the fixation point throughout the entire experiment. The reaches had a slightly larger amplitude than in the fMRI experiment (∼12.5 cm). Participants could not see their reaching hand at any point during the trials.

### Task Procedures

Participants’ task was to respond as fast and accurately as possible to the appearance of a visuospatial target. In the Arm condition, they had to respond by reaching and touching the left or the right target with their right index finger. In the Finger condition, they had to respond by releasing either the right index finger or the right middle finger from the buttons they were pressing. For every participant, the left target was associated with the release of the index finger, while the right target was associated with the release of the middle finger. All participants underwent a practice session before the experiment to ensure that movements were produced accurately without breaking fixation. Participants were also reminded between the runs that reaching movements needed to be fast and accurate.

At a variable interval before target onset (see below), an effector precue was provided to instruct participants of the upcoming effector to be used. It consisted of a 200 ms auditory tone. A high pitch tone instructed an upcoming arm reaching movement, while a low pitch tone instructed an upcoming finger movement. Concurrent with the effector precue, a spatial precue was also provided for 200 ms, allowing to manipulate participants’ knowledge of upcoming target location. In the Spatial condition, either the left or the right target was flashed in green for 200 ms, thus being fully informative as to the upcoming target location. A white target was systematically presented at the opposite target location to balance the amount of visuospatial information provided in each visual hemifield. In the Non-Spatial condition, both targets were flashed in green simultaneously, thus being non-informative as to the side on which the upcoming target would be presented.

Before each trial, participants were required to bring their right index and middle fingers to the starting base and press the button box switches, while gazing at the fixation point. If participants failed to press both switches a trial would not start, allowing us to maintain some control over the flow of the experiment. In the fMRI experiment, the duration of this baseline period varied between 2, 4, or 6 s, jittered using an m-sequence (levels = 3; power = 4) to ensure optimal statistical power of the general linear model (GLM) (Buracas and Boynton, 2002; Liu, 2004). In the EEG experiment, the duration of the baseline period was 2 s. After the baseline period, the effector and spatial precues were provided for 200 ms. After a variable delay (2.2, 2.4, or 2.6 s; pseudo-randomized), the target was presented and participants performed the task (arm reaching or finger movement). The target disappeared upon movement initiation (as recorded by the release of the button box switches). In the Arm condition, participants were told to produce straight and smooth movements toward the targets, to remain stationary for a brief moment at the end of the movement (∼1 s), and to bring their hand back to the starting base for the next trial. The out-and-back movement was generally achieved in less than 2 s. In the Finger condition, participants simply had to release the switch for ∼1 s before pressing it back again. Additional no-go trials were added in the fMRI experiment (8% of trials), which consisted of presenting a red target. Participants had to refrain from executing the planned movement in these trials. Their main purpose was to increase the efficiency of the design matrix, as well as to maintain participants’ vigilance. It was a priori determined that they would not considered for further analysis.

The fMRI experiment comprised five runs of 108 trials each, for a total of 540 trials. The trials for each condition of interest were evenly distributed across the runs. There were 124 trials in each of the 2-Effector (Arm, Finger) × 2-Precue (Spatial, Non-Spatial) factorial design as well as 44 no-go trials. Two 30 s breaks were provided within each run to allow participants to rest. The total time of the experiment was ∼70 min. The sequence of presentation of the trials as well as the orthogonality of the design matrices were assessed prior to data collection to ensure adequate power to detect blood oxygenated level dependent (BOLD) activations for all conditions of interest.

The EEG experiment comprised eight separate runs. Each run consisted of 100 trials, for a total of 800 trials. The trials for each condition of interest were pseudo-randomized and evenly distributed across the runs. There were 200 trials in each of the 2-Effector (Arm, Finger) × 2-Precue (Spatial, Non-Spatial) factorial design. A 30 s break was provided in the middle of each run to allow participants to rest. The total time of the experiment was ∼80 min.

To study arm-target integration during reach planning, one possibility would have been to directly compare neural activity in the Spatial Arm and Non-Spatial Arm conditions. However, spatial cueing inevitably introduces a shift in visuospatial attention, with participants attending to the left or right targets during the delay period in the Spatial Arm condition but not in the Non-Spatial Arm condition. This makes it difficult to unpack the cortical processes associated with arm-target integration from those associated with shifts in visuospatial attention. Hence the Spatial Arm and Non-Spatial Arm conditions were not contrasted together, but rather against the Spatial Finger and Non-Spatial Finger conditions, respectively. The latter were identical in every respect, with the only exception that participants did not have to reach to the targets, thereby isolating the arm-target integration process necessary to define a motor goal. Hence, for both fMRI and EEG, neural activity associated with spatial reach planning was obtained by contrasting the Spatial Arm and Spatial Finger conditions, whereas neural activity associated with non-spatial reach preparation was obtained by contrasting the Non-Spatial Arm and Non-Spatial Finger conditions. Given that the requirement for a speeded response was common to the two effectors, these contrasts effectively controlled for the sensory (visual) aspects of the task, for visuospatial attention (Colby and Goldberg, 1999), as well as any temporal anticipation signal that could increase as a function of the delay with the approaching time to move (Cisek et al., 2009; Saleh et al., 2010; Kaufman et al., 2016).

### Behavioral Analyses

Reaction time (RT) was defined as the time between target onset and movement onset, as measured by the release of the button box switches. RTs below 150 ms and above 600 ms were discarded (3% of data). Incorrect responses (i.e., arm movement instead of finger movement or vice-versa, movement of the incorrect finger) were determined through the button box and removed from further analyses (4% of data). Although movement kinematics were not recorded in the present experiments, debriefing with participants revealed that they had no problem complying with the requirement to reach toward the appearing visual target.

In both the fMRI and EEG experiments, there were no significant RT differences as a function of Target Laterality (*p* > 0.05), hence the two levels of this factor (Left, Right) were pooled for further analyses. RT data were thus submitted to a 2-Effector (Arm, Finger) × 2-Precue (Spatial, Non-Spatial) repeated-measures analysis of variance (ANOVA). The threshold for statistical significance was set to *p* < 0.05.

### MRI Scanning and Analyses

Functional MRI data were acquired using a Siemens 3T Magnetom TIM Trio system with a 12 channel phased-array head coil. For each functional run, a T2^∗^-weighted echo planar gradient-echo imaging (EPI) sequence sensitive to blood oxygenated level dependent (BOLD) contrast was acquired [repetition time (TR) = 2000 ms; echo time (TE) = 30 ms; flip angle (FA) = 90°; field of view (FOV) = 192 mm × 192 mm]. Each volume consisted of 37 slices acquired parallel to the AC-PC plane (interleaved acquisition; 3 mm with 0.5 mm gap; 3 mm × 3 mm in-plane resolution). Before the functional runs, a high-resolution T1-weighted sagittal sequence image of the whole brain was acquired (TR = 15 ms; TE = 4.2 ms; FA = 9 degrees; FOV = 256 mm).

Functional MRI data preprocessing and statistical analyses were carried out in SPM8^1^. The first three functional volumes of each run were automatically removed by the scanner operating system software to eliminate non-equilibrium effects of magnetization, prior to the start of the task. Individual scans were spatially realigned to the middle image of the time-series, slice-time corrected, registered to the anatomical image and normalized to Montreal Neurological Institute (MNI) space (resampled at 3 mm × 3 mm × 3 mm resolution). Images were temporally high-pass filtered with a 128 s cutoff. The functional data were then smoothed with an 8-mm full width half-maximum isotropic Gaussian kernel. Finally, even with the head perfectly stable, the dislocation of a mass near, but outside the head coil can induce signal changes in the images. A weighted least-squares algorithm was thus used to weigh each image by the inverse of its variance, therefore minimizing the impact of outlier images in the estimation of the GLM (Diedrichsen and Shadmehr, 2005).

First-level whole-brain fMRI analyses, estimated for each participant individually, sought to identify brain regions active during the preparatory delay period. Using a standard GLM, the fMRI time-series was fitted with six regressors (Spatial Arm Left, Spatial Arm Right, Spatial Finger Left, Spatial Finger Right, Non-Spatial Arm, and Non-Spatial Finger). The regressors consisted of a boxcar function with preparatory delay period as duration (the time between precue onset and target onset, which could be either 2.2, 2.4, or 2.6 s in duration). Four regressors for neural activity taking place during RT were also used (Arm Left, Arm Right, Finger Left, and Finger Right), but not analyzed further. They consisted of a boxcar function with RT as duration (the time between target onset and movement onset). Separate regressors of non-interest were also added to account for no-go trials, error trials, the effect of multiple scanning runs and MR drift. Each event was convolved with the standard gamma-shaped HRF.

For each participant, brain areas active during the preparatory delay period were first assessed for each of the six Effector-Precue-Target conditions by contrasting these conditions to baseline. The influence of Target Laterality in the Spatial conditions was then assessed by separately contrasting Spatial Arm Left vs. Spatial Arm Right, and Spatial Finger Left vs. Spatial Finger Right. Given the absence of a significant effect of Target Laterality in either effector task in the parieto-frontal cortex (see Results), data were pooled across targets for further analyses.

To specifically isolate reach-related activations, the Arm conditions were contrasted to the Finger conditions. The main contrasts were Spatial Arm > Spatial Finger to assess spatial reach planning, and Non-Spatial Arm > Non-Spatial Finger to assess non-spatial reach preparation. These contrasts were performed using an inclusive mask of regions showing significant activity in Spatial Arm > baseline or Non-Spatial Arm > baseline, respectively. This was done to ensure that the differential activations obtained in the two main Arm > Finger contrasts would occur in regions that showed a significant positive BOLD response as compared to baseline, and would not be merely due to negative BOLD responses in the Finger conditions (which there were none; see Results).

A second-level random-effects analysis was then applied to individual contrasts of parameter estimates to obtain a population estimate. All reported statistics are corrected for False Discovery Rate at *q*(FDR) < 0.05 (Genovese et al., 2002).

For visualization purposes, the *t*-images were mapped to the partially inflated cortical surface of the Population Average Landmark and Surface-based (PALS-B12) atlas (Van Essen, 2005) using the Caret software application. The PALS-B12 atlas represents the surface registration of 12 normal adult high-resolution scans, which can be used as an unbiased template for displaying images from group fMRI analyses.

The main aim of the fMRI analysis was to directly compare the parieto-frontal activations during spatial reach planning (i.e., Spatial Arm > Spatial Finger) and non-spatial reach preparation (i.e., Non-Spatial Arm > Non-Spatial Finger). This was done using a region of interest (ROI) analysis. Five regions lying along the dorsal visual stream and implicated in reach planning were selected. These were the bilateral medial bank of the intraparietal sulcus (mIPS), located half-way up the length of the intraparietal sulcus (Gallivan et al., 2011; Bernier et al., 2012; Fabbri et al., 2014; Haar et al., 2015), the posterior part of the medial bank of the IPS (pIPS; Beurze et al., 2009; Gallivan et al., 2011; Bernier et al., 2012; Fabbri et al., 2014), the medial and anterior part of the parieto-occipital sulcus (parieto-occipital junction, POJ; Gallivan et al., 2011), the dorsal part of the PMd, located at the junction of the precentral sulcus and superior frontal sulcus (Bernier and Grafton, 2010; Gallivan et al., 2011; Bernier et al., 2012), and the primary motor cortex (M1; Gallivan et al., 2013). To ensure independent ROI definition, the peak coordinates were taken from previous studies from our laboratory (except for M1), which used an identical experimental setup and a similar task. For mIPS, pIPS, and PMd, the coordinates were taken from Bernier et al. (2012). The MNI coordinates were [-30, -54, 60] and [27, -54, 66] for left and right mIPS, respectively, [-18, -69, 54] and [18, -66, 60] for left and right pIPS, respectively [labeled dorsomedial PPC in Bernier et al. (2012)], and [-27, -12, 60] and [24, -15, 63] for left and right PMd, respectively [labeled PMd caudal in Bernier et al. (2012)]. The POJ coordinates were taken from Bernier and Grafton (2010) who also used a similar experimental setup. They were [-14, -84, 38] and [20, -72, 36] for left and right hemispheres, respectively. The M1 coordinates were taken from Gallivan et al. (2013) and were [-32, -26, 59] and [29, -26, 59] for left and right hemispheres, respectively. The ROIs were 10 mm-radius spheres around these peak coordinates. These independent coordinates revealed to be similar to the local maxima observed in these regions in the current main contrasts Spatial Arm > Spatial Finger and Non-Spatial Arm > Non-Spatial Finger.

To statistically compare activity within these ROIs, data from the five regions were submitted to separate 2-Hemisphere (Left, Right) × 2-Precue (Spatial, Non-Spatial) repeated-measures ANOVAs (after having confirmed the normality of the data with the Shapiro–Wilk test). Upon a significant interaction, paired-samples *t*-tests were used to compare the different levels of the two factors. The threshold for statistical significance was set to *p* < 0.05, adjusted for multiple comparisons using Bonferroni correction.

### EEG Data Acquisition and Analyses

Electroencephalography was acquired using a 64-channel MR-compatible BrainAmp system (Brain products, Munich, Germany), along with the BrainCap electrode cap (Falk Minow Services, Herrsching-Breitbrunn, Germany). When placing the cap, we made sure that the Cz electrode was at the vertex. The electrodes were ring-type sintered non-magnetic Ag/AgCl electrodes and were positioned in accordance with the extended 10/20 system. The reference electrode was located between Fz and Cz. Vertical eye movements and blinks were monitored with frontal electrode FP1 (positioned above the left orbit). The EEG signals were digitized online (sampling rate 5 kHz), and impedances were kept below 20 kΩ.

Electroencephalography data were analyzed off-line using the Brain Vision Analyzer software (version 2.0; Brain products, Munich, Germany). Data were down-sampled to 200 Hz. They were digitally bandpass filtered off-line (0.5–50 Hz, 12 dB/octave) and transformed to the average reference (Gwin et al., 2010; Gwin and Ferris, 2012a,b). Using data from channel FP1, trials in which saccadic eye movements occurred during the preparatory delay or the RT interval were removed (<1% of trials for all participants). Participants were encouraged to delay their blinks until the inter-trial interval, yet remaining ocular artifacts were subtracted from the EEG signal using the statistical method of Gratton et al. (1983). The data were also visually inspected for stereotypical artifacts such as excessive peak-to-peak deflections, or bursts of EMG activity. The data were epoched within a window between -4200 and +1800 s around target onset for all conditions. They were then exported into the EEGLAB toolbox (Delorme and Makeig, 2004) in Matlab (MathWorks, Natick, MA, USA) for further analyses.

In EEGLAB, the data were further inspected for artifacts with a procedure based on independent component analysis (ICA), a standard method for removal of artifacts from EEG (Makeig et al., 2002; Delorme and Makeig, 2004; Hammon et al., 2008; Gwin et al., 2010; Gwin and Ferris, 2012a,b). The ‘runica’ procedure in EEGLAB was applied to decompose EEG signals into statistically maximally independent components (ICs). ICs were analyzed with respect to scalp topography and frequency characteristics, and those that displayed features indicative of artifacts were removed. Cleaned EEG data were generated by projecting back the time course of activity of the remaining ICs to the electrodes. This procedure allows the removal of artifacts from the EEG without having to reject the entire trial during which an artifact occurred (Jung et al., 2000; Whittingstall et al., 2010).

Monopolar EEG recordings were transformed to current source density (CSD) estimates using Laplacian transformation as implemented in the CSD toolbox (Kayser and Tenke, 2006) in MATLAB. The signal was interpolated with a spherical spline interpolation procedure (Perrin et al., 1987) to compute second-order derivatives in two dimensions of space (order of splines: 3; maximal degree of Legendre polynomials: 10; approximation parameter λ: 1.0e–005). CSD data are much less affected by far-field generators than monopolar recordings (Manahilov et al., 1992), thus enhancing the spatial and temporal resolution of the recordings (Burle et al., 2015; Vidal et al., 2015).

To assess changes in spectral power during the preparatory delay, time varying event-related spectral perturbations (ERSP) were estimated using EEGLAB (Delorme and Makeig, 2004). This was done by convolving each participant’s single trial data with a sinusoidal wavelet before averaging across trials. Each trial was partitioned into 200 segments and power was analyzed between 3 and 40 Hz using a sliding window technique. The number of cycles for the lowest frequency was set to 3 and slowly increased with frequency (factor 0.2), allowing better frequency resolution at higher frequencies. ERSPs were investigated in the alpha-band (8–12 Hz) and the beta-band (15–30 Hz) in three temporal epochs spanning the preparatory delay period: (1) Early planning (-2000 to -1500 ms before target onset), Mid planning (-1250 to -750 ms before target onset) and Late planning (-500 to 0 ms before target onset). Event-related desynchronization (ERD) values were calculated for each epoch and each frequency band. They were obtained by subtracting the mean power during baseline, which was defined as the period between -4200 and -2700 ms before target onset, from the mean power in each epoch. This baseline period was chosen because participants were motionless and because the effector and spatial precues were not presented yet. Hence, brain activity was considered neutral at that moment since no planning could be going on.

To visually appreciate the scalp distribution of alpha- and beta-band ERD, topographic maps were created using the mean alpha and beta ERD values for each epoch at each electrode. Because alpha- and beta-band ERD could be modulated by Target Laterality (Left, Right) in the Spatial Arm and Spatial Finger conditions, data were first contrasted across the two targets separately for each Effector (Spatial Arm Left vs. Spatial Arm Right and Spatial Finger Left vs. Spatial Finger Right). This was done using paired-samples *t*-tests at each electrode, corrected for multiple comparisons (62 electrodes) using Bonferroni correction. Since there was no significant modulation of Target Laterality in either frequency band (see Results), the data were pooled across the two target dimensions for further analyses.

Similar with the fMRI analyses, the main EEG analyses sought to identify the oscillatory activities associated with spatial reach planning and non-spatial reach preparation. To isolate spatial reach planning activities, alpha- and beta-band ERD values in the Spatial Arm condition were contrasted to those in the Spatial Finger condition. To isolate non-spatial reach preparation activities, alpha- and beta-band ERD values in the Non-Spatial Arm condition were contrasted to those in the Non-Spatial Finger condition. Differences across Effectors (Arm vs. Finger) were statistically assessed using paired-samples *t*-tests at each electrode (Bonferroni-corrected), separately for the three temporal epochs. Topographic maps of the statistical differences in alpha- and beta-band ERD at each epoch were then created by plotting the corrected *t*-scores for each electrode.

The main aim of the EEG analysis was to directly compare the alpha- and beta-band ERD values during spatial reach planning and non-spatial reach preparation. This was done using a ROI analysis. This analysis was carried out only on data from the Late planning epoch, since it was assumed that the time-window just prior to the anticipated moment of target onset would most faithfully capture the reach preparatory processes across the different conditions. Specifically, ROIs were defined over bilateral parietal/parieto-occipital electrodes (P1, P3, PO3 and P2, P4, PO4 for left and right hemispheres, respectively) as well as bilateral central/fronto-central electrodes (C1, C3, FC1, FC3 and C2, C4, FC2, FC4 for left and right hemispheres, respectively; see inset of **Figure 7** for location of electrodes). To statistically compare activity within these ROIs, the difference in alpha- and beta-band ERD across Effectors (Arm minus Finger) were submitted to separate 2-Hemispheres (Left, Right) × 2-Precues (Spatial, Non-Spatial) repeated-measures ANOVAs (after having confirmed the normality of the data with the Shapiro–Wilk test). Upon a significant interaction, paired-samples *t*-tests were used to compare the different levels of the two factors. The threshold for statistical significance was set to *p* < 0.05, adjusted for multiple comparisons using Bonferroni correction.

Finally, in light of previous work showing a greater dependency between preparatory activity in the PRR and RT during non-spatial reach preparation as compared to spatial reach planning (Snyder et al., 2006), a correlation analysis was used to assess a possible relationship between preparatory activity in the alpha- and beta-bands and behavior. Specifically, for each condition and each participant, a Spearman rank correlation was performed between single-trial ERD values in the Late planning epoch and single-trial RTs. The correlations at each electrode were then averaged across participants. To provide a whole-brain depiction of potential differences in correlations, a topographic map of the difference in correlations across Effectors (Arm vs. Finger) was done using paired-samples *t*-tests at each electrode. Then, to statistically assess differences within each of the four ROIs (left and right parietal/parieto-occipital; left and right central/fronto-central), the correlation coefficients were submitted to a 2-Effector (Arm, Finger) × 2-Precue (Spatial, Non-Spatial) repeated-measures ANOVA, separately for the alpha- and beta-bands (after having confirmed the normality of the data with the Shapiro–Wilk test). Upon a significant interaction, paired-samples *t*-tests were used to compare the different levels of the two factors. The threshold for statistical significance was set to *p* < 0.05, adjusted for multiple comparisons using Bonferroni correction.

## Results

### Reaction Time

The RT data from the fMRI and EEG experiments are presented in **Table 1**. Despite differences in the experimental setups, the results were qualitatively similar. In the fMRI experiment, the ANOVA revealed a significant main effect of Precue [*F*_(1,15)_ = 36.4; *p* < 0.001]. Participants were slower to initiate their responses in the Non-Spatial conditions (mean RT for Non-Spatial Arm and Non-Spatial Finger = 435 ms) than in the Spatial conditions (mean RT for Spatial Arm and Spatial Finger = 364 ms). In the EEG experiment, the ANOVA revealed a significant main effect of Precue [*F*_(1,13)_ = 80.8; *p* < 0.001], with participants responding slower in the Non-Spatial conditions (mean RT for Non-Spatial Arm and Non-Spatial Finger = 346 ms) than in the Spatial conditions (mean RT for Spatial Arm and Spatial Finger = 299 ms). The ANOVA also revealed an interaction between Effector and Precue [*F*_(1,13)_ = 19.9; *p* < 0.01]. Specifically, RT was similar across Effectors in the Non-Spatial conditions (346 ms for Arm and 346 ms for Finger), but slightly faster for Finger than Arm in the Spatial conditions (292 ms vs. 307 ms, respectively). This is possibly due to a postural dependency, with simpler biomechanics involved in initiating a finger movement as compared to an arm movement, being easier to detect during upright EEG recording than supine fMRI.

**Table 1:** Mean reaction times in each condition for the fMRI and electroencephalography (EEG) experiments.

	Condition	Reaction times (ms)
fMRI experiment	Non-Spatial Arm	416 ± 21
	Non-Spatial Finger	453 ± 24
	Spatial Arm	350 ± 24
	Spatial Finger	379 ± 28
EEG experiment	Non-Spatial Arm	346 ± 11
	Non-Spatial Finger	346 ± 10
	Spatial Arm	307 ± 10
	Spatial Finger	293 ± 11

*Values are ±SE*.

### fMRI (Whole-Brain Analysis)

The first fMRI analysis characterized brain areas recruited in the Spatial Arm and Spatial Finger conditions. The influence of Target Laterality on neural activity was first assessed separately for the Arm and Finger conditions, doing the following contrasts: Arm Left vs. Arm Right and Finger Left vs. Finger Right. In the Arm conditions, the left target yielded significantly more activity (*p* < 0.05 FDR-corrected) in the right lingual gyrus (BA 19, [30, -73, -11]) as compared to the right target. No region was more active for the right target as compared to the left target at that threshold. In the Finger conditions, the left target incurred significantly more activity in the right inferior occipital gyrus (BA18; [30, -82, -8]), whereas the right target incurred more activity in the left lingual gyrus (BA18; [-27, -82, -11]; both *p* < 0.05 FDR-corrected). In sum, given that Target Laterality only had an influence on visual areas lying outside of the parieto-frontal regions that were the main focus of the this work, data from the two targets were pooled for further analyses.

The areas showing a significant BOLD response (*p* < 0.05 FDR-corrected) in the Spatial Arm and Spatial Finger conditions with respect to baseline were first assessed. Arm reach planning incurred significant activity in much of the parieto-frontal network, including the left sensorimotor cortex, bilateral superior parietal lobule (SPL), bilateral inferior parietal lobule (IPL), bilateral middle frontal gyrus (i.e., PMd and PMv), the supplementary motor area (SMA), bilateral putamen, left inferior frontal gyrus, the thalamus and the right cerebellum. In contrast, finger movement planning was mainly restricted to the frontal lobe, with a significant BOLD response in left sensorimotor cortex, bilateral PMd and PMv, SMA and slight activity in left SPL. No region showed a negative BOLD response in either condition.

**FIGURE 2:**
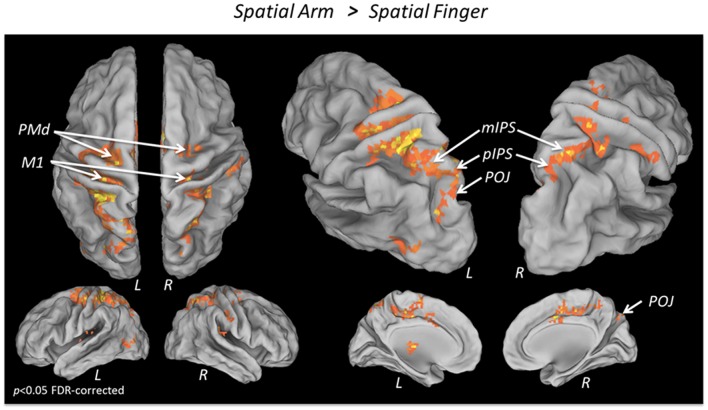
**fMRI BOLD response during Spatial reach planning**. Areas showing a significantly greater BOLD response in the Spatial Arm as compared to the Spatial Finger condition, overlaid on an inflated cortical surface (*p* < 0.05 FDR; no significant activation for the reverse contrast Spatial Finger > Spatial Arm).

To isolate the activations specifically related to spatial reach planning, the contrast Spatial Arm > Spatial Finger was performed (**Figure 2** and **Table 2**). The Spatial Arm condition incurred significantly greater activity (*p* < 0.05 FDR-corrected) in bilateral primary motor cortex (M1), bilateral PMd as well as along the entire medial bank of the intraparietal sulcus bilaterally, including the anterior (aIPS), medial (mIPS), and posterior parts (pIPS). Activity was also observed in the POJ bilaterally. There was also greater activity in the postcentral gyrus bilaterally (BA 3, BA 5, and BA 40), in the anterior cingulate and SMA as well as in the posterior lobe of the right cerebellum. Although activation was observed bilaterally in the PPC and PMd, there was a tendency toward greater contralateral (left) recruitment in these regions (see “ROI analysis” for further analyses).

**Table 2:** Average MNI coordinates (in mm) of clusters showing significantly more activity in the Spatial Arm as compared to the Spatial Finger condition.

	Anatomical region	Functional label (Brodmann area)	Hemisphere	*x*	*y*	*z*	*t*-value
Spatial Arm > Spatial Finger	Precentral gyrus	PMd (BA 6)	Left	-21	-19	70	9.50
	Superior frontal gyrus	PMd (BA 6)	Right	18	-1	67	8.98
	Precentral gyrus	M1 (BA 4)	Left	-30	-28	67	10.63
	Precentral gyrus	M1 (BA 4)	Right	18	-28	64	6.35
	Medial frontal gyrus	SMA (BA 6)	Left	-3	-4	55	9.24
	Cingulate gyrus	Anterior cingulate (BA 32)	Right	6	2	43	8.20
	Postcentral gyrus	BA 3/BA 5/BA 40	Left	-27	-40	58	8.55
	Postcentral gyrus	BA 3	Right	24	-31	64	8.82
	Superior parietal lobule	aIPS (BA 7)	Left	-30	-43	67	9.61
	Superior parietal lobule	aIPS (BA 7)	Right	30	-49	55	6.04
	Superior parietal lobule	mIPS (BA 7)	Left	-24	-58	58	9.46
	Superior parietal lobule	mIPS (BA 7)	Right	27	-58	61	9.25
	Superior parietal lobule	pIPS (BA 7)	Left	-18	-67	58	7.90
	Superior parietal lobule	pIPS (BA 7)	Right	15	-67	58	4.99
	Parieto-occipital junction	POJ (BA 19)	Left	-15	-82	37	5.81
	Parieto-occipital junction	POJ (BA 19)	Right	6	-79	43	6.31
	Cerebellum	Posterior lobe (vermis)	Right	6	-64	-20	7.94

The second fMRI analysis characterized the brain areas showing a significant BOLD response in the Non-Spatial Arm and Non-Spatial Finger conditions with respect to baseline. The Non-Spatial Arm condition incurred activity in largely the same set of regions as in the Spatial Arm condition, including bilateral sensorimotor cortex, bilateral PMd, SPL, IPL as well as SMA, bilateral putamen, thalamus and right cerebellum. Again, the Non-Spatial Finger condition incurred activity in a much more restricted set of areas almost exclusively in the frontal lobe, including the left sensorimotor cortex, bilateral middle frontal gyrus (PMd), left SPL and SMA. No region showed a negative BOLD response in either condition.

**FIGURE 3:**
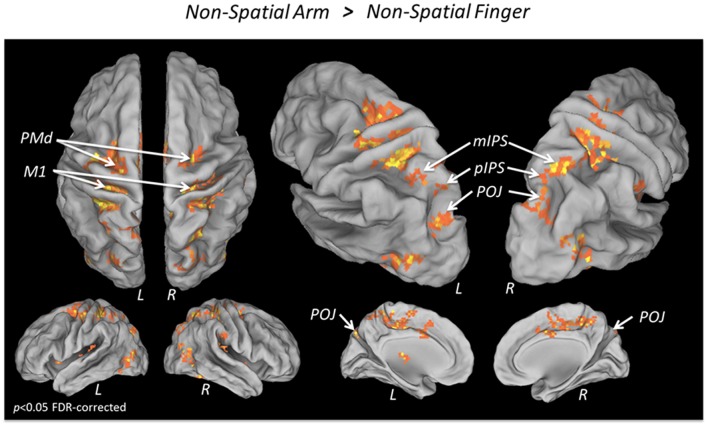
**fMRI BOLD response during Non-spatial reach preparation**. Areas showing a significantly greater BOLD response in the Non-Spatial Arm as compared to the Non-Spatial Finger condition, overlaid on an inflated cortical surface (*p* < 0.05 FDR; no significant activation for the reverse contrast Non-Spatial Finger > Non-Spatial Arm).

To isolate the activations specifically related to non-spatial reach preparation, we performed a contrast between the Non-Spatial Arm and Non-Spatial Finger conditions (**Figure 3** and **Table 3**). Greater non-spatial reach preparatory activity was observed in most of the same regions as for spatial reach planning, including bilateral M1, bilateral PMd and the entire intraparietal sulcus bilaterally, with clusters in the aIPS, mIPS, pIPS, and POJ. There was also greater activity in the postcentral gyrus bilaterally, in the anterior cingulate and SMA as well as the right cerebellum. Interestingly, in contrast to spatial reach planning, non-spatial reach preparation incurred activity in the PPC much more bilaterally.

**Table 3:** Average MNI coordinates (in mm) of clusters showing significantly more activity in the Non-Spatial Arm as compared to the Non-Spatial Finger condition.

	Anatomical region	Functional label (Brodmann area)	Hemisphere	*x*	*y*	*z*	*t*-value
**Non-Spatial Arm > Non-Spatial Finger**	Middle frontal gyrus	PMd (BA 6)	Left	-24	-13	52	8.24
	Middle frontal gyrus	PMd (BA 6)	Right	24	-7	61	8.98
	Precentral gyrus	M1 (BA 4)	Left	-30	-31	58	9.04
	Precentral gyrus	M1 (BA 4)	Right	30	-28	57	8.40
	Postcentral gyrus	S1 (BA 1)	Right	21	-34	70	9.47
	Medial frontal gyrus	SMA (BA 6)	Right	3	-13	55	8.22
	Cingulate gyrus	Anterior cingulate (BA 32)	Right	3	5	46	7.80
	Postcentral gyrus	BA 40/BA 3	Left	-33	-40	58	8.75
	Postcentral gyrus	BA 40/BA 3	Right	27	-37	61	9.04
	Superior parietal lobule	aIPS (BA 7)	Left	-21	-49	55	6.03
	Superior parietal lobule	aIPS (BA 7)	Right	27	-49	55	5.13
	Superior parietal lobule	mIPS (BA 7)	Left	-21	-58	61	7.81
	Superior parietal lobule	mIPS (BA 7)	Right	24	-58	55	9.41
	Superior parietal lobule	pIPS (BA 7)	Left	-15	-67	55	7.50
	Superior parietal lobule	pIPS (BA 7)	Right	24	-73	49	5.59
	Parieto-occipital junction	POJ (BA 19)	Left	-15	-82	40	7.92
	Parieto-occipital junction	POJ (BA 19)	Right	24	-79	37	6.08
	Cerebellum	Posterior lobe (vermis)	Right	3	-67	-20	5.50

### fMRI (ROI Analysis)

**FIGURE 4:**
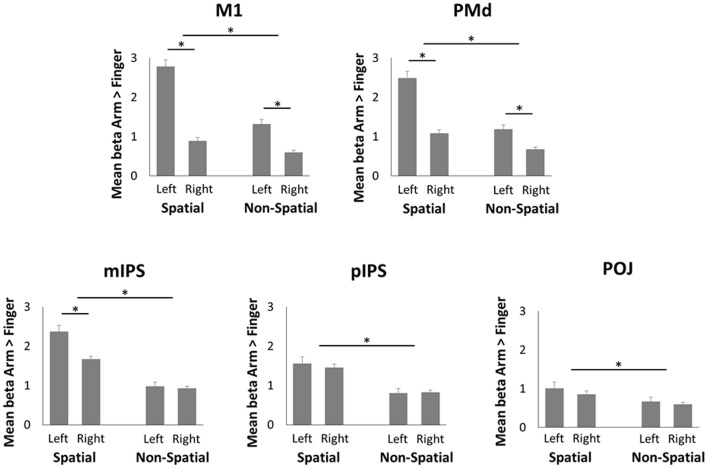
**Region of interest (ROI) analysis in the fMRI experiment**. Mean beta values during Spatial reach planning (Spatial Arm > Spatial Finger) and Non-Spatial reach preparation (Non-Spatial Arm > Non-Spatial Finger) in bilateral M1, PMd, mIPS, pIPS, and POJ. Asterisks denote a significant difference at *p* < 0.05.

To compare neural activity in the parieto-frontal network during spatial reach planning and non-spatial reach preparation, ROIs were created in bilateral M1, PMd, mIPS, pIPS, and POJ, the results of which are presented in **Figure 4**. The pattern of results that emerged for M1 and PMd was similar. Specifically, the ANOVAs revealed a significant main effect of Hemisphere [*F*_(1,15)_ = 97.3 and *F*_(1,15)_ = 30.6 for M1 and PMd, respectively, both *p* < 0.001], with greater activity in the left (contralateral) hemisphere as compared to the right hemisphere. There was also a significant main effect of Precue [*F*_(1,15)_ = 144.0 and *F*_(1,15)_ = 72.5 for M1 and PMd, respectively, both *p* < 0.001], with more activity in the Spatial than Non-Spatial condition. Finally, the ANOVAs also revealed a significant interaction [*F*_(1,15)_ = 132.4 and *F*_(1,15)_ = 40.3 for M1 and PMd, respectively, both *p* < 0.001], with the interhemispheric difference in activity being greater in the Spatial than Non-Spatial condition. Paired-samples *t*-tests revealed that activity was greater in the left than right hemisphere in both the Spatial and Non-Spatial conditions (*p* < 0.001 Bonferroni-corrected). In the mIPS, the ANOVA also revealed a significant main effect of Hemisphere [*F*_(1,15)_ = 6.7; *p* < 0.05], a significant main effect of Precue [*F*_(1,15)_ = 68.0; *p* < 0.001] and a significant interaction [*F*_(1,15)_ = 14.7; *p* < 0.01]. Paired-samples *t*-tests revealed that activity was greater in the left than right hemisphere in the Spatial condition (*p* < 0.05 Bonferroni-corrected), but did not differ across hemispheres in the Non-Spatial condition (*p* = 0.55). Finally, in the pIPS and POJ, the ANOVAs only revealed a significant main effect of Precue [*F*_(1,15)_ = 28.4 and *F*_(1,15)_ = 6.9 for pIPS and POJ, respectively, both *p* < 0.01], with activity being greater in the Spatial as compared to the Non-Spatial condition. Importantly, activity did not differ across hemispheres in either the Spatial or Non-Spatial conditions (confirmed by paired-samples *t*-tests, all *p* > 0.4). In sum, the ROI analysis revealed an interaction in mIPS, with activity being bilateral during non-spatial reach preparation, but left lateralized during spatial reach planning.

### EEG (Whole-Brain Analysis)

After removal of the artifacts, an average of 724 ± 48 epochs per participant were retained across the different conditions. The number of trials in each Effector and Precue condition did not differ significantly (*p* > 0.5).

The first main EEG analysis identified differences in oscillatory activity in two bandwidths associated with spatial reach planning, tested across the three temporal epochs. Results revealed that no electrode was significantly modulated by Target Laterality in either the alpha- or the beta-band (*p* < 0.05 Bonferroni-corrected). Hence the data were pooled across the two levels of this factor for further analyses.

**FIGURE 5:**
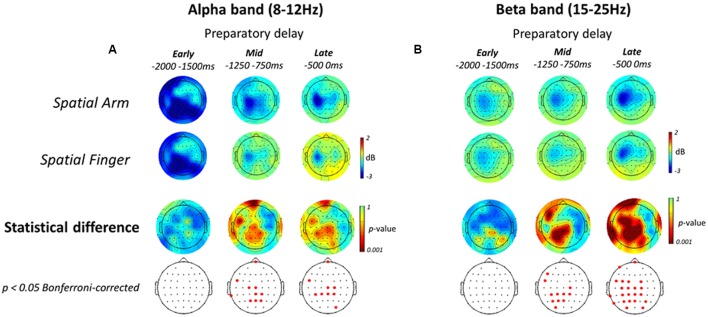
**Electroencephalography alpha- and beta-band ERD during Spatial reach planning**. (**A**, top two rows) Scalp topographic maps of alpha-band ERD in the Early, Mid, and Late planning epochs of the preparatory delay period, shown for the Spatial Arm and Spatial Finger conditions. (**A**, bottom two rows) Scalp topographic maps of the statistical difference in alpha-band ERD between the Spatial Arm and Spatial Finger conditions, along with the electrodes that survive a *p* < 0.05 Bonferroni-corrected criterion. **(B)** Same as **(A)** but for beta-band ERD.

The topographic maps associated with alpha and beta-band ERD in the Spatial Arm and Spatial Finger conditions for each epoch are presented in **Figures 5A,B**. As can be seen, both conditions were associated with alpha-band ERD over bilateral parietal electrodes in the Early planning epoch, which then shifted toward left sensorimotor electrodes in the Mid and Late planning epochs (**Figure 5A**, top two rows). In the beta-band, the Spatial Arm and Spatial Finger conditions were associated with gradually increasing ERD peaking over the left sensorimotor regions (**Figure 5B**, top two rows).

To isolate the oscillatory activities specifically associated with spatial reach planning, the power in each band was compared across the two Effectors (Arm vs. Finger). The statistical topographic maps of the electrodes that differed significantly at a *p* < 0.05 Bonferroni-corrected threshold are presented in the bottom two rows of **Figures 5A,B**. In the alpha-band, there was no difference across Effectors in the Early planning epoch. In the Mid planning epoch, alpha-band ERD became significantly greater in the Spatial Arm condition at central, centro-parietal and parietal electrodes. A similar pattern was observed in the Late planning epoch, with significant differences at central and centro-parietal electrodes.

In the beta-band, there was no difference across Effectors in the Early planning epoch. Then, in Mid planning, beta-band ERD became prominent over the left centro-parietal and parietal electrodes. Finally, in Late planning, beta-band ERD became significantly greater over the entire left parieto-frontal cortex, at parieto-occipital, parietal, centro-parietal, central and fronto-central electrodes. In sum, the process of representing a motor goal during spatial reach planning was associated with increasing beta-band ERD over parietal and precentral regions, predominantly in the left (contralateral) hemisphere.

**FIGURE 6:**
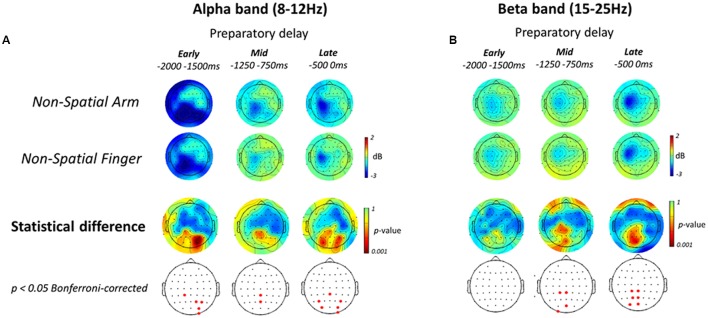
**Electroencephalography alpha- and beta-band ERD during Non-spatial reach preparation**. (**A**, top two rows) Scalp topographic maps of alpha-band ERD in the Early, Mid, and Late planning epochs of the preparatory delay period, shown for the Non-Spatial Arm and Non-Spatial Finger conditions. (**A**, bottom two rows) Scalp topographic maps of the statistical difference in alpha-band ERD between the Non-Spatial Arm and Non-Spatial Finger conditions, along with the electrodes that survive a *p* < 0.05 Bonferroni-corrected criterion. **(B)** Same as **(A)** but for beta-band ERD.

The second main EEG analysis pertained to non-spatial reach preparation, as presented in **Figure 6**. Similar to the Spatial conditions, in both Effector conditions alpha-band ERD was prominent over bilateral parietal electrodes in the Early planning epoch, and then shifted toward the left sensorimotor regions (**Figure 6A** top two rows). Also similar to the Spatial conditions, in both Effector conditions beta-band ERD gradually increased throughout the preparatory delay period, peaking over the sensorimotor regions in the left hemisphere (**Figure 6B** top two rows).

The patterns of ERD associated with non-spatial reach preparation, resulting from the contrast between the Non-Spatial Arm and Non-Spatial Finger conditions, are presented in the bottom two rows of **Figures 6A,B**. In the alpha-band, non-spatial reach preparation was associated with activity predominantly at right parietal and parieto-occipital electrodes in the Early planning epoch, and activity at centro-parietal scalp sites in Mid planning. Interestingly, in contrast to what was observed for spatial reach planning, non-spatial reach preparation in the Late planning epoch was associated with activity at bilateral parietal and parieto-occipital scalp sites.

As for the beta-band, the Early planning epoch revealed no significant difference across Effector conditions. Differences became prominent in Mid planning, with significant activity in the left centro-parietal and parieto-occipital electrodes. Ultimately, in the Late planning epoch, differences remained localized in contralateral parietal regions, specifically at left centro-parietal, parietal and parieto-occipital electrodes. In sum, contrary to the widespread parieto-frontal activities observed for spatial reach planning, oscillatory activities during non-spatial reach preparation was restricted to parietal scalp sites: bilateral alpha-band ERD and contralateralized beta-band ERD.

### EEG (ROI Analysis)

**FIGURE 7:**
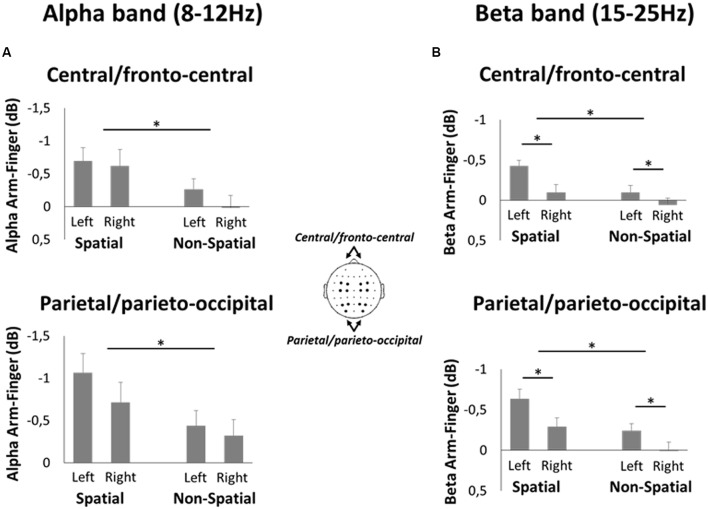
**Region of interest analysis in the EEG experiment**. **(A)** Mean difference in alpha-band ERD during Spatial reach planning (Spatial Arm minus Spatial Finger) and Non-Spatial reach preparation (Non-Spatial Arm minus Non-Spatial Finger) at bilateral central/fronto-central electrodes (top panel) and parietal/parieto-occipital electrodes (bottom panel). **(B)** Same as A but for beta-band ERD. Asterisks denote a significant difference at *p* < 0.05.

To directly compare the alpha- and beta-band activities during the Late planning epoch between spatial reach planning and non-spatial reach preparation, a ROI analysis was performed (**Figure 7**). Specifically, ROIs were defined in bilateral parietal/parieto-occipital regions as well as bilateral central/fronto-central regions. The pattern of results that emerged for the alpha-band was similar across the two regions. Specifically, there was a significant main effect of Precue [*F*_(1,13)_ = 7.6 and *F*_(1,13)_ = 7.5 for parietal/parieto-occipital regions and central/fronto-central regions, respectively, both *p* < 0.05], with alpha-band ERD being greater in the Spatial than Non-Spatial condition. Importantly, however, there was no significant main effect of Hemisphere nor an interaction, suggesting that alpha-band activity was bilateral. As for the beta-band, again the two regions showed a similar pattern of results. Specifically, there was a significant main effect of Precue [*F*_(1,13)_ = 13.6 and *F*_(1,13)_ = 6.3 for parietal/parieto-occipital regions and central/fronto-central regions, respectively, both *p* < 0.05], with beta-band ERD being greater in the Spatial condition. Furthermore, there was a main effect of Hemisphere [*F*_(1,13)_ = 13.7 and *F*_(1,13)_ = 26.5 for parietal/parieto-occipital regions and central/fronto-central regions, respectively, both *p* < 0.01], suggesting that beta-band activity was greater in the left (contralateral) hemisphere. There was no significant interaction in either region.

### EEG (ERD-RT Correlation)

**FIGURE 8:**
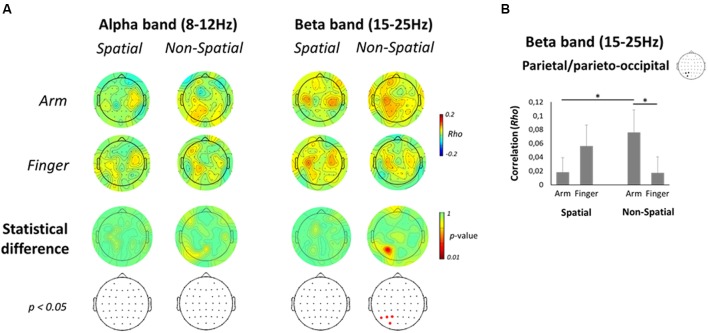
**Correlation between EEG activity and RT**. (**A**, top two rows) Scalp topographic maps of correlation coefficients between alpha- (left) and beta-band (right) single-trial ERD values in the Late planning epoch and RTs, presented separately for each Effector and Precue condition. (**A**, bottom two rows) Scalp topographic maps of the statistical difference in correlation coefficients between the Arm and Finger conditions, along with the electrodes that survive a *p* < 0.05 criterion. **(B)** Mean correlation coefficient between beta-band ERD and RT for each Effector and Precue condition in the left parietal/parieto-occipital ROI. Asterisks denote a significant difference at *p* < 0.05.

To assess a potential relationship between oscillatory activities in the Late planning epoch and behavior, a correlation was performed between single-trial ERD values in the Late planning epoch and RTs. As can be seen on **Figure 8A** (top two rows), the correlations, albeit low, tended to be highest (∼0.15) at central and parietal electrodes. The positive correlations indicate that the greater the ERD in the Late planning epoch, the faster the RT. In the alpha-band, no electrode presented a significant difference as a function of effector in either the Spatial or Non-Spatial condition (**Figure 8A**, bottom two rows). In contrast, in the beta-band, electrodes overlaying the left PPC (P1, P3, P5, and PO3) presented a significantly greater correlation with RT in the Non-Spatial Arm condition as compared to the Non-Spatial Finger condition. Interestingly, this effect was not observed in the Spatial conditions. Statistical analysis of the ROI data confirmed the above observations. Indeed, the left parietal/parieto-occipital ROI presented a significant interaction [*F*_(1,13)_ = 8.6; *p* < 0.01; see **Figure 8B**]. Breakdown of this interaction using paired-samples *t*-tests revealed that correlations were significantly higher in Non-Spatial Arm (*rho* = 0.08) as compared to Non-Spatial Finger (*rho* = 0.02; *p* < 0.01). Importantly, correlations were also higher in Non-Spatial Arm as compared to Spatial Arm (*rho* = 0.02; *p* < 0.05). These data argue for a stronger relationship between beta-band ERD in contralateral PPC and motor behavior specifically when a reaching movement is prepared in absence of target information. None of the other ANOVAs conducted on the four ROIs for both frequency bands revealed a significant effect.

## Discussion

The goal of the present study was to compare reach-related neural activity in the parieto-frontal cortex when upcoming target location is either specified in advance or not. By focusing on differential activations between arm and finger movements, the experimental design allowed us to isolate processes associated with reaching. In contrast to spatial reach planning, which induced activity across parietal and frontal regions primarily contralateral to the reaching arm, non-spatial reach preparation was associated with BOLD responses bilaterally in the PPC. Furthermore, alpha- and beta-band activity in the non-spatial condition was restricted to parietal scalp sites, the magnitude of the latter being correlated with reach RTs. These qualitative differences as a function of spatial cueing argue for an intermediate stage of sensorimotor transformations in bilateral parietal cortex when target location is not specified.

### Spatial Reach Planning Engages Parieto-Frontal Regions Contralateral to the Arm

During spatial reach planning (Spatial Arm > Spatial Finger), BOLD responses were found to be stronger in the mIPS, PMd, and M1 contralateral to the reaching arm. This is consistent with previous reports in both monkeys and humans, showing arm selectivity in these regions when a motor goal can be formed (Snyder et al., 1997; Connolly et al., 2003; Beurze et al., 2007; Chang et al., 2008; Beurze et al., 2009; Van Der Werf et al., 2010; Gallivan et al., 2011; Heed et al., 2011; Vesia and Crawford, 2012; Leone et al., 2014; Cappadocia et al., 2016). The pattern of beta-band ERD provided additional insight as to the temporal evolution of motor goal formation. Indeed, beta-band ERD increased over the course of the delay period, being observed first over parietal electrodes and then more broadly over parietal and frontal electrodes prior to movement onset. Activity was also contralateralized with respect to the arm, suggesting the encoding of the reach plan in coordinates tied to the effector. Overall these data replicate the known involvement of contralateral parietal and frontal regions during standard sensorimotor transformations.

### Non-spatial Reach Preparation Incurs Bilateral Activity in PPC

One of the main findings of the present work is the interaction between hemisphere and spatial cueing observed in the mIPS. Indeed, unlike the contralateral arm selectivity for spatial reach planning, non-spatial reach preparation (Non-Spatial Arm > Non-Spatial Finger) was associated with a bilateral BOLD response in the mIPS. The lack of a contralateral dominance in this context may seem paradoxical, given that the arm to be used was also known in advance. The bilateral response suggests a stage of sensorimotor processing that acted upstream from arm-target integration. One possibility is that it reflected the active representation of the two visuospatial targets, which were presented in both the left and right hemifields, before either of them was confirmed as the actual target [see also Bernier et al. (2012)]. This would be supported by the finding that alpha-band ERD was also observed bilaterally at parieto-occipital electrodes during non-spatial reach preparation. Given that alpha-band activity over the PPC has been shown to contribute to the internal representation of spatial goals relative to gaze (Van Der Werf et al., 2010; Buchholz et al., 2013), the bilateral pattern of ERD suggests the active maintenance of the two targets.

An alternative possibility is that the bilateral PPC activity during the delay was not related to the retrospective encoding of the two visuospatial targets, but was rather linked to visuospatial attention, in anticipation of the target being presented in either hemifield (Worden et al., 2000; Thut et al., 2006). In this scheme, the alpha-band ERD may have served to facilitate the processing of upcoming visual signals by increasing the excitability of parieto-occipital regions (Worden et al., 2000; Sauseng et al., 2005; Romei et al., 2008), without necessarily encoding the visuospatial targets *per se*. While the present data cannot disentangle between these possibilities, it should be noted that all activations reported in the present work result from a contrast between the arm and finger tasks, which largely controlled for visuospatial attentional demands. Indeed, both tasks required participants to attend to the targets in order to rapidly initiate the arm or finger responses, as indirectly confirmed by the comparable RTs across conditions. In this light, the increased activity in the PPC during arm as compared to finger movement preparation (both in BOLD and EEG) is unlikely to be merely accounted for by a general visual attentional phenomenon. Rather, it points to a role of the PPC in processing visual signals specifically when they constitute spatial goals for upcoming reaching movements, but not when they instruct upcoming movements «arbitrarily» (i.e., index or middle finger). This interpretation is consistent with findings from Baldauf et al. (2008), who showed that PRR cells do not respond to visual stimuli when they act as go-cues, but respond strongly when these same stimuli act as spatial goals for reaching movements.

### Non-spatial Reach Preparation Incurs Beta-Band ERD Only at Parietal Electrodes, Which Correlates with RT

In contrast to spatial reach planning, which induced beta-band ERD over the entire parieto-frontal network, non-spatial reach preparation induced beta-band ERD in a much more circumscribed manner only over the contralateral PPC, even late in the preparatory delay period. This also supports an intermediate stage of reach preparation in which downstream frontal regions are not engaged to the same extent as when a motor goal can be specified. Given the contralateral bias and the fact that beta-band ERD over the SPL has been suggested to reflect somatosensory processing in body-centered coordinates (Buchholz et al., 2011, 2013), it is possible that this activity was related to the proprioceptive encoding of the initial posture of the arm, in preparation of the upcoming arm-target integration process. This activity may have been subtended by SPL neuronal populations showing contralateral somatosensory responses (Breveglieri et al., 2006), which have been shown to maintain postural representations of the upper limb specifically for the planning of reaching movements (Pellijeff et al., 2006).

Interestingly, beta-band ERD in the left parieto-occipital electrodes presented a significantly stronger correlation with RT in the Non-Spatial Arm condition as compared to the Non-Spatial Finger condition. This demonstrates that preparatory activity in the PPC just prior to movement onset was linked to motor output, and more so in the context in which a reaching movement was being prepared. This is consistent with the present BOLD and alpha-band results, pointing to a specific role of the PPC for reaching as compared to finger movements. The specificity of the RT effect to reaching also indirectly suggests that the beta-band modulations over this region reflected arm and not target encoding, since a preparatory signal related to the latter would have been expected to similarly influence the arm and finger conditions.

Most importantly, the correlation was also significantly stronger in the Non-Spatial Arm condition as compared to the Spatial Arm condition. Hence pre-stimulus beta-band ERD in the PPC was related to reach onset only when the visuospatial target was not known in advance. This pattern of results is identical to that reported by Snyder et al. (2006), who showed that delay period spiking activity in PRR predicts contralateral reach RT selectively when target location is unknown, and not when it is specified in advance. The dependency of RT upon beta-band ERD only during non-spatial reach preparation further points to an intermediate stage of sensorimotor transformations, as further processing (i.e., arm-target integration process) had to be achieved after target onset, and the level of preparation as indexed by beta-band ERD influenced how quickly it could be achieved. In contrast, the weaker correlation during spatial reach planning may result from the fact that in this context a motor goal could be formed in advance, such that the target did not carry novel information and merely acted as a go-cue. All in all, the present results suggest a potential link between preparatory beta-band activity in the PPC and the heightened baseline firing in PRR previously observed in similar contexts.

### Encoding of the Timing of Movement Onset?

An alternative hypothesis is that non-spatial reach preparatory activities were associated with movement timing rather than the encoding of the spatial features of the upcoming movement. Specifically, it is possible that some of the preparatory activity was accounted for by an anticipatory signal related to the upcoming transition to movement. This view is supported by recent work from Kaufman et al. (2016) who recorded neuronal activity in M1 and PMd neurons during reach planning. They showed that many neurons demonstrated robust responses that were “condition-invariant”: their magnitude and time course were nearly identical regardless of reach direction. Moreover, even though individual M1 and PMd neurons reflected the motor goal, at a population level they more strongly reflected the timing of movement onset rather than movement type. Nevertheless, it should be noted that in the present work the variable duration of the preparatory period limited the possibility for such temporally locked activity. Furthermore, any process related to timing would likely be equated by virtue of the contrast between the arm and finger tasks, which were subject to the same temporal pressure. Finally, while such timing activity has been shown in M1 and PMd, it is less clear whether it also accounts for activity in higher-order regions such as the PPC (Michaels et al., 2015; Ohata et al., 2016).

### Representing Multiple Motor Goals in Parallel?

Current models posit that the dorsal parieto-frontal cortex encodes target locations directly in the form of potential motor goals, with the maintenance of multiple motor goals in parallel serving as the basis for selection (Baldauf et al., 2008; Cisek and Kalaska, 2010; Christopoulos et al., 2015; but see Haith et al., 2016). As such, reach preparation with target uncertainty has been shown to be associated with lower spike rates in M1 and PMd (Cisek and Kalaska, 2005; Churchland et al., 2008) as well as lower EEG or MEG responses over the sensorimotor cortex (Praamstra et al., 2009; Tzagarakis et al., 2010, 2015). Such findings have been used to support the parallel planning hypothesis, with lower activity being attributable to mutually suppressive interactions between co-existing motor goals. Although the present results are not incompatible with this interpretation, the fact that the spatio-temporal pattern of BOLD and EEG responses not only differed in terms of amplitude but also qualitatively across the Spatial and Non-Spatial conditions argues against the simultaneous encoding of two motor goals during non-spatial reach preparation. A more parsimonious explanation may be that if indeed there was parallel encoding, it occurred at a sensory level, through the simultaneous representation of the two visuospatial targets in bilateral PPC. In this light, non-spatial reach preparation may represent a form of anticipatory activity in which the brain is “preparing-to-integrate” by actively maintaining the necessary postural and visuospatial signals until a target is unambiguously specified and a motor goal can be formed.

## Author Contributions

P-MB performed experiments. P-MB analyzed data. P-MB and SG interpreted results of experiments. P-MB prepared figures. P-MB, SG, and KW edited and revised manuscript. P-MB, SG, and KW approved final version of manuscript. P-MB and SG conception and design of research. P-MB drafted manuscript.

## Conflict of Interest Statement

The authors declare that the research was conducted in the absence of any commercial or financial relationships that could be construed as a potential conflict of interest.

## Footnotes

1 www.fil.ion.ucl.ac.uk/spm
